# Technical and clinical success of endoscopic hand-suturing: a European multicenter study

**DOI:** 10.1055/a-2909-3681

**Published:** 2026-09-30

**Authors:** Mousa Ayoub, Esma Z. Kızılaslan, Sandra Nagl, Fatih Aslan, Torsten Beyna, Oscar Cahyadi, Seung-Hun Chon, Werner Dolak, Jacek Drzewiecki, Peter Ewald, Stefan K. Gölder, Przemysław Kasprzyk, Arjun D. Koch, Konstantinos Kouladouros, Raúl Honrubia López, Annemijn D.I. Maan, Pedro De María, Khanh Do-Cong Pham, Daniel R. Quast, Pedro J. Rosón, Enrique Rodriguez de Santiago, Sofia P. Carbonell, Markus W. Scheppach, Michal Spychalski, Shunya Takayanagi, Christian Rafael Torres Reyes, Thomas Veiser, Benjamin M. Walter, Jochen Weigt, Christoph Zeidler, Helmut Messmann, Alanna Ebigbo

**Affiliations:** 1 Department of GastroenterologySt. Josef-Hospital Bochum, Ruhr University BochumBochumGermany; 2 III. Medical Department 39694 University Hospital AugsburgAugsburgGermany; 3 Gastroenterology 587267 Koc University HospitalIstanbulTurkey; 4 Department of Gastroenterology and HepatologyEvangelisches Krankenhaus DüsseldorfDüsseldorfGermany; 5 Department of General, Visceral, Thoracic, and Transplant SurgeryUniversity Hospital of CologneCologneGermany; 6 Department of Gastroenterology and HepatologyUniversity Hospital of CologneCologneGermany; 7 Internal Medicine III, Div. of Gastroenterology and HepatologyMedical University of ViennaViennaAustria; 8 Department of Internal Medicine and GastroenterologyProvincial Integrated HospitalElblagPoland; 9 Department of General, Gastroenterological and Oncological SurgeryCollegium Medicum Nicolaus Copernicus UniversityTorunPoland; 10 Department of Gastroenterology, Hematology/Oncology and Pulmonology 39481 Ostalb Hospital AalenAalenGermany; 11 Endoscopy and Bariatric Surgery CenterBrzeziny Specialist HospitalŁódźPoland; 12 Department of Gastroenterology and HepatologyErasmus MC Cancer InstituteRotterdamNetherlands; 13 Central Inderdisciplinary Endoscopy, Department of Hepatology and Gastroenterology 14903 Charite University Hospital BerlinBerlinGermany; 14 Department of Gastroenterology 161633 Hospital Universitario Infanta SofiaSan Sebastian de los ReyesSpain; 15 Department of Medicine, School of Medicine, Health and Sports 16740 Universidad Europea de Madrid Campus de Villaviciosa de OdónMadridSpain; 16 Department of Digestive DiseasesHospital University of La PazMadridSpain; 17 Department of MedicineHaukeland University HospitalBergenNorway; 18 Department of GastroenterologyQuirón HospitalMálagaSpain; 19 Gastroenterology and HepatologyHospital Universitario Ramón y Cajal, Universidad de Alcalá, IRYCISMadridSpain; 20 Gastroenterology and HepatologyHospital Universitario Ramón y Cajal. Universidad de Alcalá, IRYCISMadridSpain; 21 Endoscopy and Bariatric Surgery CenterBrzeziny Specialist HospitalBrzezinyPoland; 22 Department of Internal Medicine 37808 Medical University of LodzŁódźPoland; 23 Department of Internal Medicine IUniversity Hospital UlmUlmGermany; 24 Department of Gastroenterology, Hepatology and Infectious diseasesOtto-von-Guericke UniversitiyMagdeburgGermany

## Abstract

**Background:**

Endoscopic closure techniques are essential for management of endoscopic defects and help prevent delayed bleeding and perforation. This study reports clinical outcomes of an endoscopic hand-suturing system (EHS) for defect closure.

**Methods:**

This multicenter European registry study evaluated all procedures using EHS from 14 centers. The primary end point was technical success, defined as complete defect closure without additional closure techniques. Secondary end points were in-hospital clinical success (absence of post-interventional complications), overall defect closure rate (closure achieved with additional devices), and suturing speed.

**Results:**

Between February 2023 and January 2026, 338 procedures were included. Indications comprised endoscopic submucosal dissection (n = 240), endoscopic intermuscular dissection (n = 32), gastric peroral endoscopic myotomy (n = 41), fistula (n = 10), endoscopic mucosal resection (n = 6), submucosal tunneling endoscopic resection (n = 3), nonexposed endoscopic wall inversion surgery (n = 2), and anastomotic leak, perforation, endoscopic full-thickness resection, and endoscopic hemostasis (all n = 1). Median defect size was 35 mm (range 4–155 mm) and median suturing time was 25 minutes (range 4–120). Technical success was 96.2% (325/338). Overall defect closure rate was 96.7% (327/338). In-hospital clinical success was 97.9% (331/338). Defect size did not predict suturing time in the size-adjusted model, indicating that procedural complexity was not explained by size alone. Procedure-related adverse events occurred in 5/338 (1.5%); overall adverse event rate was 10/338 (3.0%).

**Conclusion:**

EHS demonstrated high technical and in-hospital clinical success with a low short-term adverse event rate. Further studies are needed to assess performance with complex defects.

## Abstract (de)

**Graphical abstract:**

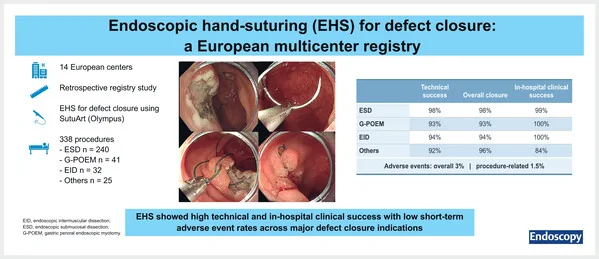

## Introduction

Advanced therapeutic endoscopy creates intentional mucosal entry sites and large post-interventional defects that can lead to clinically relevant adverse events. In some third-space procedures, secure closure of the mucosal entry site is mandatory. In these cases, defect size, location, geometry, and tissue tension after extensive resection can exceed the capabilities of simple hemostatic clip-based approximation 1 2 3 .

A broad range of endoscopic defect management strategies is available, including through-the-scope (TTS) clips, over-the-scope (OTS) clips, endoloop/clip-based combination techniques, endoscopic suturing, endoscopic stenting, and endoluminal vacuum therapy 4 5 6 7 . European guidance for iatrogenic perforations reflects this graded approach, recommending endoscopic closure as the first-line method when feasible, with modality selection guided by defect size and clinical context 8 9 .

However, each modality has practical limitations. TTS clips are fast and widely accessible but their use can be limited by defect size, edge separation under tension, and tangential access. OTS clips extend the closure range but may be restricted by access, maneuverability, and challenging anatomy. While stents and endoluminal vacuum therapy are essential tools for selected indications (e.g. leaks/cavities requiring source control), they represent a different paradigm from single-session mucosal defect closure and may entail serial endoscopic interventions and substantial resource utilization.

In this landscape, endoscopic suturing is attractive because it enables tension-bearing, edge-to-edge approximation, potentially providing a more stable construct when clipping approaches are challenged by defect size, geometry, tension, or location (e.g. duodenum) 10 11 . The most established reference platform is the Apollo OverStitch Endoscopic Suturing System (Apollo Endosurgery, Austin, Texas, USA) 12 , an OTS device mounted on a compatible endoscope that places sutures via a needle-driver mechanism. A newer single-channel-compatible iteration (OverStitch Sx) 13 has received regulatory clearance and aims to broaden compatibility and improve handling. However, OTS systems require device attachment and remain challenging in anatomically restricted segments.

TTS tissue approximation systems have emerged to simplify closure workflows and avoid bulky OTS assemblies. One such platform is the X-Tack Endoscopic HeliX Tacking System (Apollo Endosurgery), in which multiple helical tacks are sequentially anchored into healthy tissue adjacent to a defect and then approximated by tightening the connecting suture and deploying a cinch.

The SutuArt system (Olympus Medical Systems Corp., Tokyo, Japan) represents a different TTS concept. It is a rotatable TTS needle holder that enables stepwise needle control (grasping, tissue penetration, release, and regrasping) by rotating the instrument and coordinating movements with the endoscope tip ( Fig. 1 ) 14 . Sequential bites can thereby be placed on opposing wound edges under endoscopic visualization until closure is achieved, which may be advantageous in angulated anatomy.

**Fig. 1:**
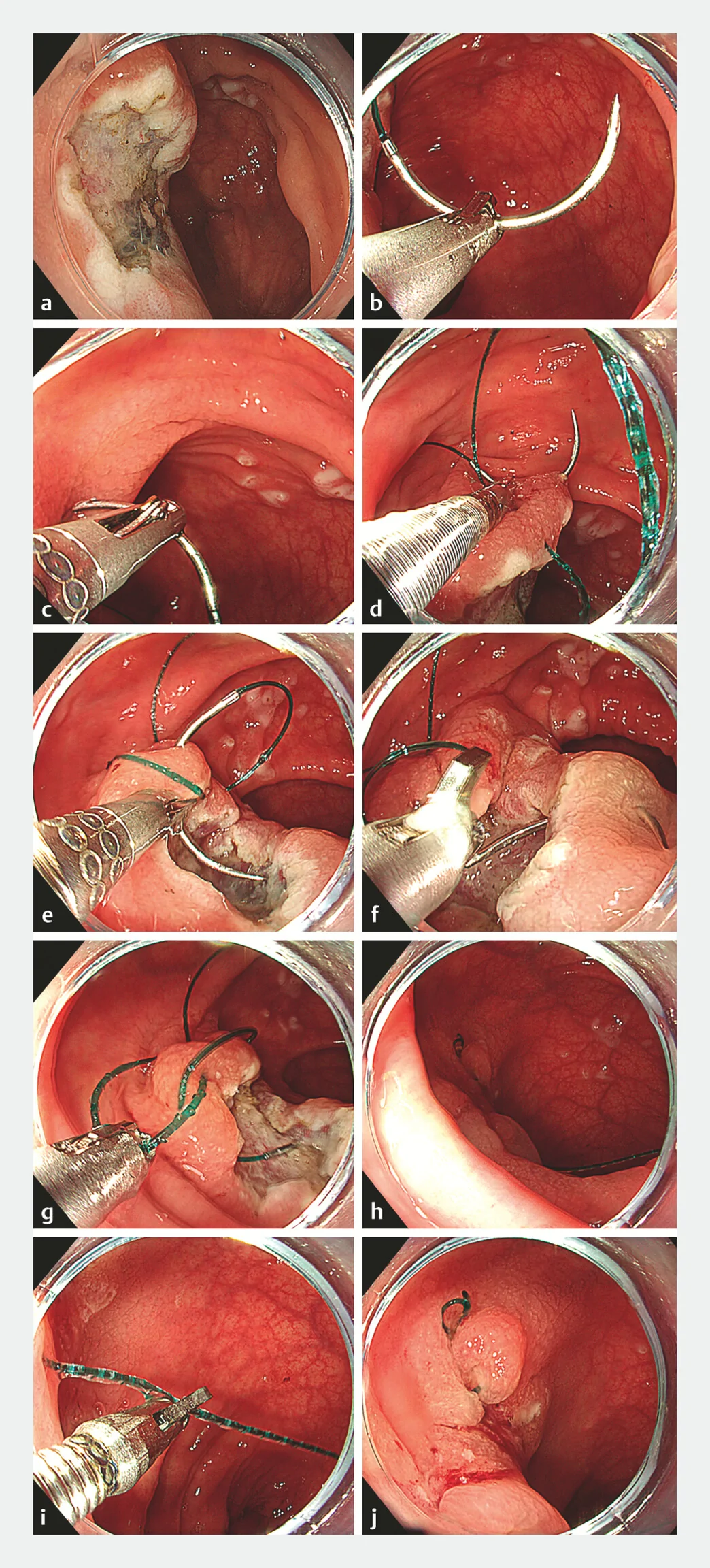
Stepwise through-the-scope (TTS) hand-suturing with SutuArt (Olympus Medical Systems Corp., Tokyo, Japan) for post-endoscopic submucosal dissection (ESD) defect closure. **a** Post-ESD mucosal defect prior to closure. **b** The curved needle is grasped with the TTS needle holder. **c** Needle entry at the targeted mucosal edge. **d** The needle is advanced and pulled through the mucosa to complete the first bite. **e,f** The needle is regrasped, repositioned, and passed through the opposing mucosal edge to complete the second bite. **g** The suture thread is grasped and the knot is tightened under endoscopic visualization. **h** Close-up view of the completed knot with the remaining suture tail. **i** The residual thread is cut using the dedicated TTS cutting step. **j** Final view demonstrating complete defect closure after hand-suturing.

Conceptually, the potential advantage of endoscopic hand-suturing (EHS) is that it combines needle-based, tension-bearing approximation with preserved endoscope maneuverability in a TTS format. In contrast, TTS clips are fast and widely available but their use is limited in larger or high-tension defects; X-Tack relies on a tacking-and-cinch mechanism rather than true needle-based suturing; and OverStitch offers durable full-thickness suturing at the cost of a more platform-dependent OTS setup.

To date, prospective multicenter evidence on the technical role and efficiency of EHS in routine clinical practice remains limited 15 16 17 18 . Therefore, we report outcomes from a European multicenter SutuArt registry evaluating the real-world performance of endoscopic defect closure.

## Methods

### Study design

This was a multicenter retrospective European registry study. Procedures performed at 14 European centers between February 2023 and January 2026 were eligible. In routine practice, case selection for EHS was based on investigator discretion rather than a standardized or randomized allocation strategy, according to defect size, geometry, access, orientation, or the anticipated feasibility of clip-based closure.

The following procedures were eligible for inclusion: 1) mural or transmural defects requiring endoscopic closure and treated with EHS; 2) peroral endoscopic myotomy (POEM) procedures in which the mucosal entry or submucosal tunnel was closed using EHS; 3) other indications at the investigator’s discretion, provided the defect was managed with the EHS system.

### Data collection and variables

The registry captured baseline and procedural variables, including age, sex, American Society of Anesthesiologists classification, indication, anatomical location, defect size (mm), intervention time (minutes), suturing time (minutes), use of antithrombotic therapy at the time of the procedure, and history of prior surgery. The defect size was assessed by the endoscopist, based on the size of the resected specimen or its relative size compared with a snare.

Primary analyses were performed according to intervention type. For additional descriptive subgroup analyses, procedures were secondarily grouped by procedural context into resection-related defects (endoscopic submucosal dissection [ESD], endoscopic intermuscular dissection [EID], endoscopic mucosal resection, endoscopic full-thickness resection) and mucosotomy/tunnel-entry closures (gastric POEM [G-POEM], submucosal tunneling endoscopic resection).

### End points and definitions

The primary end point was immediate technical success, defined as complete defect closure achieved by suturing alone, without any additional closure technique. Secondary end points were as follows: 1) in-hospital clinical success, defined as the absence of defect-related complications, such as bleeding, perforation, or infection, during the index hospital stay; post-discharge events were not prospectively captured in a standardized manner across all centers and were recorded only if reported back to the treating institution; 2) overall defect closure rate, defined as closure achieved by suturing combined with adjunctive devices (e.g. clips); 3) suturing time, recorded in minutes from first needle deployment to cutting the thread and adjusted to defect size (mm/min) to support comparability across defect sizes; device preparation time and accessory exchanges were excluded from suturing time; 4) adverse event rate according to the American Society for Gastrointestinal Endoscopy (ASGE) adverse event lexicon 19 .

### Statistical analysis

Analyses were descriptive and no formal hypothesis testing was planned. Categorical variables are reported as counts and proportions. The association between defect size and suturing time was explored descriptively using linear regression, with R ^2^ (coefficient of determination) used as a measure of model fit.

For binomial outcomes (technical success, overall defect closure, in-hospital clinical success, and adverse event rates), results are presented as n/N (%) with 95%CIs calculated using the Wilson method to provide interval estimates without implying comparative inference.

Continuous variables are summarized as mean (SD), median (Q1, Q3) and additionally reported with range (min–max) where informative. For key continuous outcomes (suturing time and size-adjusted performance metrics), 95%CI of the mean was calculated using standard parametric methods (t-based CI). To account for heterogeneity in defect size, procedural efficiency was expressed as suturing speed (mm/min) as the primary size-adjusted metric.

Adverse events were classified according to the ASGE lexicon severity grades and reported separately for overall adverse events and EHS-related adverse events, based on investigator assessment. In addition to severity-stratified counts, adverse events are reported as n/N (%) with 95%CI using the Wilson method.

### Software

Data curation and verification were performed in Microsoft Excel version 16.108.1 (Microsoft Corp., Redmond, Washington, USA). Figures were generated in GraphPad Prism version 11.0.0 (GraphPad Software, Boston, Massachusetts, USA). Manuscript-ready tables were programmatically formatted in R 4.5.2 (R Foundation for Statistical Computing, Vienna, Austria) to ensure consistent layout and typography.

### Ethics

The registry operated under central ethics committee approval from the Ethics Committee of the University Hospital Regensburg (vote no. 24–3934–101) with site-specific approvals obtained where required by national or regional regulations. All included patients provided written informed consent.

## Results

### Patient collective

A total of 338 EHS procedures were included in the analysis ( Table 1 ). Procedures included ESD (n = 240), G-POEM (n = 41), EID (n = 32), and other indications (n = 25). Among ESD cases, resections were performed in the stomach (n = 121), duodenum (n = 6), right hemicolon (n = 24), left hemicolon (n = 32), and rectum (n = 57). The “other indications” category (n = 25) comprised fistula closure (n = 10), mucosal defects after endoscopic mucosal resection (n = 6), submucosal tunneling endoscopic resection (n = 3), nonexposed endoscopic wall inversion surgery (n = 2), anastomotic leak (n = 1), perforation (n = 1), endoscopic full-thickness resection (n = 1), and endoscopic hemostasis (n = 1).

**Table:** **Table 1** Patient and procedural characteristics of 338 endoscopic hand-suturing procedures.

	N = 338
**Patient characteristics**	
Age, median (IQR) [range], years	66 (56–74) [37–91]
Sex	
Female	196 (58.0)
Male	142 (42.0)
**Antithrombotic therapy at time of procedure**	
Any antithrombotic therapy at time of procedure	88 (26.0)
DOAC	25 (7.4)
Aspirin	40 (11.8)
Dual antiplatelet therapy	8 (2.4)
P2Y12-inhibitor monotherapy	10 (3.0)
Vitamin K antagonist	1 (0.3)
Unknown regimen	4 (1.2)
**Prior major surgery**	
Any prior major surgery	30 (8.9)
Anti-reflux surgery (fundoplication)	9 (2.7)
Esophageal resection/gastric pull-up procedures	9 (2.7)
Pancreatoduodenectomy (Whipple)	1 (0.3)
Other prior surgeries	11 (3.3)
**Procedure type**	
Total procedures included	338
ESD	240 (71.0)
G-POEM	41 (12.1)
EID	32 (9.5)
Other indications	25 (7.4)
** ESD locations ^1^**	
Stomach	121 (50.4)
Duodenum	6 (2.5)
Right hemicolon	24 (10.0)
Left hemicolon	32 (13.3)
Rectum	57 (23.8)
Data are n (%) unless otherwise specified. DOAC, direct oral anticoagulant; EID, endoscopic intermuscular dissection; ESD, endoscopic submucosal dissection; G-POEM, gastric peroral endoscopic myotomy; IQR, interquartile range. ^1^ Percentages calculated based on ESD procedures (n = 240).	

Median patient age was 66 years (IQR 56–74, range 37–91); 196 (58.0%) were female and 142 (42.0%) were male. Of the 338 patients, 88 (26.0%) were receiving antithrombotic therapy, including direct oral anticoagulants (n = 25), aspirin (n = 40), dual antiplatelet therapy (n = 8), P2Y12-inhibitor monotherapy (n = 10), vitamin K antagonist (n = 1), and unknown regimen (n = 4). Six procedures were excluded because the EHS was used for indications that did not require defect closure (endoscopic transoral outlet reduction and stent fixation).

### Defect size

The median defect size was 35 mm (IQR 21.5–50), with a mean of 38.3 mm and an overall range of 4–155 mm. By indication, median defect size was 20 mm (IQR 15–20, range 12–25) for G-POEM and 40 mm (IQR 30–55, range 10–155) for ESD.

### Technical success

In the overall cohort (n = 338), technical success was achieved in 96.2% (95%CI 93.5–97.7) ( Table 2 , Fig. 2 **a** ). Technical success was 97.5% for ESD (95%CI 94.6–98.8), 92.7% for G-POEM (95%CI 80.6–97.5), 93.8% for EID (95%CI 79.9–98.3), and 92.0% for others (95%CI 75.0–97.8).

**Table:** **Table 2** Success outcomes and suturing efficiency by intervention and endoscopic submucosal dissection subgroup.

Group	n/N	Success rate, %	95%CI ^1^
Technical success ^2^			
Total	325/338	96.2	93.5–97.7
ESD	234/240	97.5	94.6–98.8
Stomach	118/121	97.5	93.0–99.2
Duodenum	5/6	83.3	43.6–97.0
Right hemicolon	24/24	100	86.2–100
Left hemicolon	30/32	93.8	79.9–98.3
Rectum	57/57	100	93.7–100
G-POEM	38/41	92.7	80.6–97.5
EID	30/32	93.8	79.9–98.3
Others	23/25	92.0	75.0–97.8
Overall defect closure rate ^3^			
Total	327/338	96.7	94.3–98.2
ESD	235/240	97.9	95.2–99.1
Stomach	119/121	98.3	94.2–99.6
Duodenum	5/6	83.3	43.6–97.0
Right hemicolon	24/24	100	86.2–100
Left hemicolon	30/32	93.8	79.9–98.3
Rectum	57/57	100	93.7–100
G-POEM	38/41	92.7	80.6–97.5
EID	30/32	93.8	79.9–98.3
Others	24/25	96.0	80.5–99.3
In-hospital clinical success ^4^			
Total	331/338	97.9	95.8–99.0
ESD	237/240	98.8	96.4–99.6
Stomach	120/121	99.2	95.5–99.8
Duodenum	6/6	100	61.0–100
Right hemicolon	24/24	100	86.2–100
Left hemicolon	31/32	96.9	84.3–99.4
Rectum	56/57	98.2	90.7–99.7
G-POEM	41/41	100	91.4–100
EID	32/32	100	89.3–100
Others	21/25	84.0	65.4–93.6
EID, endoscopic intermuscular dissection; ESD, endoscopic submucosal dissection; G-POEM, gastric peroral endoscopic myotomy. ^1^ 95%CI calculated using the Wilson method. ^2^ Primary technical success, defined as complete defect closure achieved by suturing alone without adjunctive closure devices. ^3^ Overall defect closure rate, defined as complete closure achieved by suturing with adjunctive devices. ^4^ In-hospital clinical success, defined as absence of post-interventional complications during the index hospitalization and no complications attributable to the index procedure.			

**Fig. 2:**
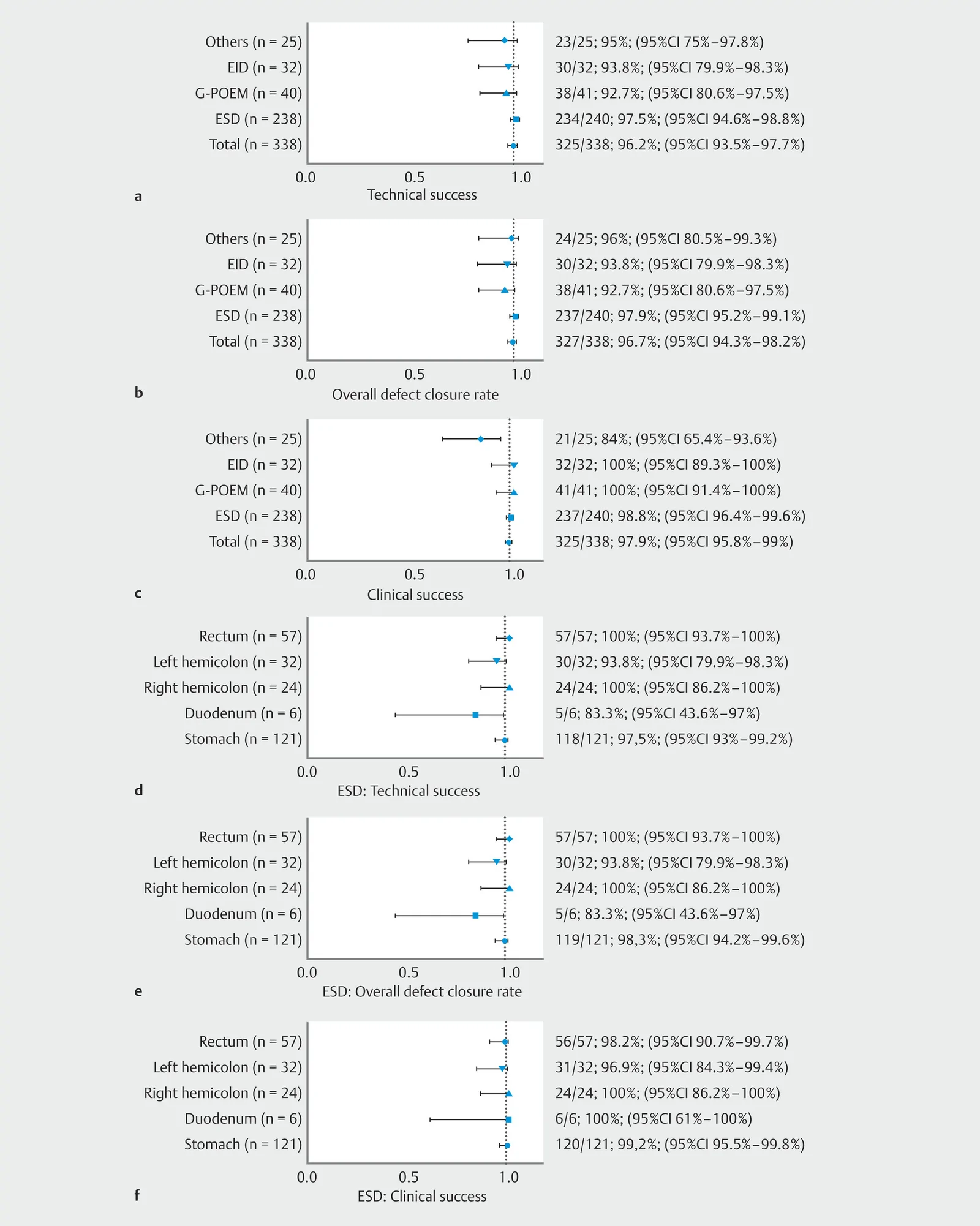
Technical and in-hospital clinical success, and overall defect closure rates by intervention and subgroups undergoing endoscopic submucosal dissection (ESD). Forest plot-style dot-and-whisker plots display proportions ( *x* -axis, 0–1) with corresponding 95%CIs (horizontal whiskers) for technical success, overall defect closure rate, and in-hospital clinical success, respectively. Categories are shown on the *y* -axis. The vertical dashed reference line indicates the respective overall rate for the comparison set: overall cohort in panels **a–c** and overall ESD rate in panels **d–f** . **a** Technical success by intervention. **b** Overall defect closure rate by intervention. **c** In-hospital clinical success by intervention. **d** Technical success within ESD, stratified by anatomical subgroup. **e** Overall defect closure rate within ESD, stratified by anatomical subgroup. **f** In-hospital clinical success within ESD, stratified by anatomical subgroup.

These pooled rates should be interpreted in the context of clinically relevant subgroup variability, with lower technical success in duodenal ESD (83.3%, 95%CI 43.6–97.0) than in gastric ESD (97.5%, 95%CI 93.0–99.2), left hemicolon ESD (93.8%, 95%CI 79.9–98.3), and right hemicolon (100%, 95%CI 86.2–100), or rectal ESD (100%, 95%CI 93.7–100) ( Fig. 2 **d** ).

### Overall defect closure rate

The overall defect closure rate was 96.7% (95%CI 94.3–98.2) ( Table 2 , Fig. 2 **b** ). Closure rates were 97.9% for ESD (95%CI 95.2–99.1), 92.7% for G-POEM (95%CI 80.6–97.5), 93.8% for EID (95%CI 79.9–98.3), and 96.0% for other interventions (95%CI 80.5–99.3).

Similarly, overall defect closure was not uniform across subgroups, with lower rates in duodenal ESD (83.3%, 95%CI 43.6–97.0) than in gastric ESD (98.3%, 95%CI 94.2–99.6), left hemicolon (93.8%, 95%CI 79.9–98.3), right hemicolon (100%, 95%CI 86.2–100), and rectal ESD (100%, 95%CI 93.7–100) ( Fig. 2 **e** ).

### In-hospital clinical success

In-hospital clinical success was observed in 97.9% (95%CI 95.8–99.0) ( Table 2 , Fig. 2 **c** ). By intervention, in-hospital clinical success was 98.8% for ESD (95%CI 96.4–99.6), 100% for G-POEM (95%CI 91.4–100) and EID (95%CI 89.3–100), and 84.0% for other interventions (95%CI 65.4–93.6). In the ESD subgroup, in-hospital clinical success reached 99.2% in the stomach (95%CI 95.5–99.8), 100% in the duodenum (95%CI 61.0–100) and right hemicolon (95%CI 86.2–100), 96.9% in left hemicolon (95%CI 84.3–99.4), and 98.2% in the rectum (95%CI 90.7–99.7) ( Fig. 2 **f** ).

### Suturing time

Across all procedures, median suturing time was 25 minutes (range 4–120). The size-adjusted suturing speed (mm/min) showed a median of 1.33 (IQR 0.89–2.42) and a mean of 1.83 (SD 1.38), with a 95%CI of the mean of 1.68–1.97 and range of 0.83–9.69 ( Table 3 , Fig. 3 **a,b** ). Median size-adjusted suturing speed by intervention type was 1.6 mm/min (IQR 1.08–3.00) for ESD, 0.63 mm/min (IQR 0.47–0.85) for G-POEM, and 1.33 mm/min (IQR 1.18–1.6) for EID.

**Table:** **Table 3** Suturing efficiency measured as suturing speed (mm/min), defined as defect size adjusted to suturing time, for the overall cohort (N = 335) and stratified by intervention type.

Group	N	Mean (SD)	Median	Q1, Q3	Range	95%CI (mean)
Total	335	1.83 (1.38)	1.33	0.89, 2.42	0.83–9.69	1.68–1.97
ESD	238	2.16 (1.47)	1.60	1.08, 3.00	0.32–9.69	1.98–2.35
G-POEM	40	0.71 (0.34)	0.63	0.47, 0.85	0.20–1.67	0.61–0.81
EID	32	1.38 (0.49)	1.33	1.18, 1.60	0.50–3.00	1.21–1.55
EID, endoscopic intermuscular dissection; ESD, endoscopic submucosal dissection; G-POEM, gastric peroral endoscopic myotomy; Q, quartile.						

**Fig. 3:**
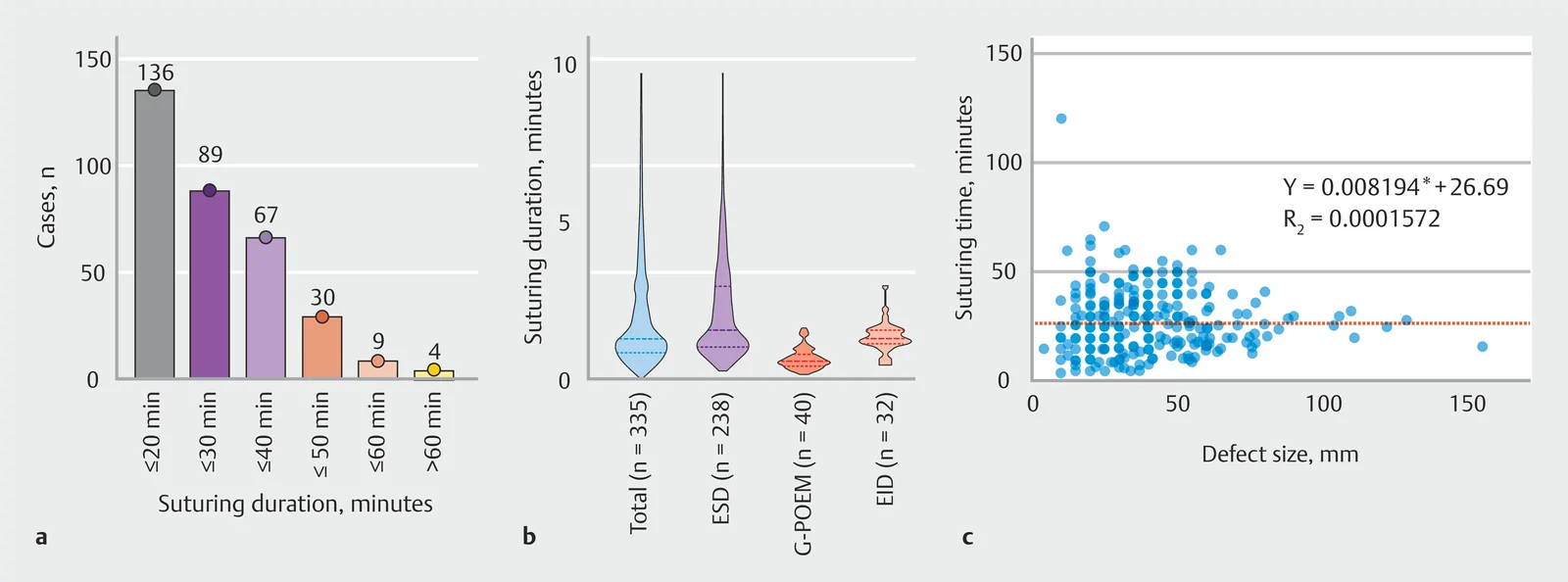
Efficiency metrics for endoscopic hand-suturing. Composite figure summarizing procedural efficiency and size-adjusted time modeling. **a** Distribution of suturing duration categories across the overall cohort, shown as number of cases per time interval. **b** Suturing efficiency expressed as suturing speed (mm/min) by intervention category, displayed as violin plots with central tendency and dispersion overlays. **c** Scatter plot of defect size (mm) versus suturing time (minutes) for the overall cohort with fitted linear regression. In the pooled cohort, the size–time association was negligible (R ^2^ = 0.0001572), indicating that defect size alone did not explain the variability in overall procedural time. EID, endoscopic intermuscular dissection; ESD, endoscopic submucosal dissection; G-POEM, gastric peroral endoscopic myotomy.

### Adverse events

Overall, 10/338 (3.0%) adverse events occurred (95%CI [Wilson] 1.62–5.36) ( Table 4 ). Adverse events were graded as mild (n = 7), moderate (n = 2), and severe (n = 1), according to the ASGE lexicon, and no fatal events occurred. Procedure-related adverse events were observed in 5/338 (1.5%) (95%CI [Wilson] 0.63–3.42), including mild (n = 2), moderate (n = 2), and severe (n = 1) events, with no fatal events. Most events were identified during the index hospitalization; however, one severe delayed bleeding event occurred after discharge.

**Table:** **Table 4Adverse events stratified by severity according to the** American Society for Gastrointestinal Endoscopy **lexicon and by procedural relatedness.**

	ASGE severity			
	Mild	Moderate	Severe	Fatal
All adverse events	N = 7	N = 2	N = 1	N = 0
Event types	Aspiration pneumonia Incomplete fistula closure on follow-up Mild post-ESD bleeding Periprocedural infection Muscular vein thrombosis with pulmonary embolism Post-procedural fever without evidence of perforation Post-procedural pain	Secondary perforation Reopening of the defect site with bleeding	Delayed bleeding after discharge with hemorrhagic shock due to suture dehiscence	–
Overall adverse event rate ^1^ : 10/338 (3.0%) [95%CI ^2^ 1.62–5.36]				
Procedure-related adverse events	N = 2	N = 2	N = 1	N = 0
Event types	Incomplete fistula closure on follow-up Mild post-ESD bleeding	Secondary perforation Reopening of the defect site with bleeding	Delayed bleeding after discharge with hemorrhagic shock due to suture dehiscence	–
Overall procedure-related adverse event rate ^1^ : 5/338 (1.5%) [95%CI ^2^ 0.63–3.42]				
ASGE, American Society for Gastrointestinal Endoscopy; ESD, endoscopic submucosal dissection. ^1^ Event rates were calculated using the total cohort as denominator (N = 338). ^2^ 95%CIs calculated using the Wilson method.				

### Procedural context analysis

To improve interpretability across the two principal procedural contexts represented in the registry, additional descriptive subgroup analyses were performed separating resection-related defects (n = 279) from mucosotomy/tunnel-entry closures (n = 44). In resection-related defects, technical success was 97.1% (271/279; 95%CI 94.4–98.5), overall defect closure was 97.5% (272/279; 95%CI 94.9–98.8), and in-hospital clinical success was 98.9% (276/279; 95%CI 96.9–99.6). In mucosotomy/tunnel-entry closures, the corresponding rates were 90.9% (40/44; 95%CI 78.8–96.4), 93.2% (41/44; 95%CI 81.8–97.7), and 100% (44/44; 95%CI 92.0–100), respectively. Median suturing speed, based on available suturing time data, was 1.50 mm/min (IQR 1.08–2.77) in resection-related defects and 0.67 mm/min (IQR 0.47–1.00) in mucosotomy/tunnel-entry closures. Overall adverse events occurred in 5/279 (1.8%) resection-related cases and 3/44 (6.8%) mucosotomy/tunnel-entry cases, whereas procedure-related adverse events occurred in 4/279 (1.4%) and 0/44 cases, respectively (see **Table 1s** in the online-only Supplementary material).

### Size–time relationship

In the overall cohort, the association between defect size and suturing time was negligible (R ^2^ = 0.0001572), indicating that defect size alone did not explain the variability in suturing time ( Fig. 3 **c** ).

## Discussion

In this European multicenter registry, EHS demonstrated high feasibility for defect closure across a broad spectrum of indications. Primary technical success (suturing alone) was high overall and remained consistently high across the main procedural categories (ESD, G-POEM, and EID), while overall closure (including adjunctive devices) approached completeness. In-hospital clinical success was similarly high, and short-term adverse events were infrequent, with few severe events and no fatal outcomes. However, these pooled outcomes should be interpreted in the context of clinically relevant subgroup heterogeneity, particularly within the ESD subgroup and the small, heterogeneous group of other indications.

Additional context-based subgroup analyses showed that in resection-related defects, technical success and overall defect closure were numerically higher, and size-adjusted suturing speed was descriptively higher in resection-related defects than in mucosotomy/tunnel-entry closures, whereas in-hospital clinical success remained high in both groups ( **Table 1s** ). A plausible explanation is that open post-resection defects may allow more direct edge identification and approximation, while mucosotomy/tunnel-entry closure, particularly in dynamic antral/pyloric settings, may require more precise bite placement and stable endoscope control despite smaller and more standardized defect geometry.

A key practical implication of the findings is that the hand-suturing approach may integrate into routine therapeutic workflows without the platform and setup constraints of bulky OTS assemblies. In daily practice, closure challenges often arise in settings combining large defects, irregular geometry, tangential approach angles, and substantial tissue tension features that can exceed the reliable range of standard clipping strategies. The consistently high technical success observed across the major indication groups suggests that EHS can provide a stable edge-to-edge approximation in clinically relevant scenarios. Importantly, in duodenal ESD, a high-risk environment for delayed events, technical success was numerically lower than in other ESD locations; however, the small case number limits inference and underscores the need for cautious interpretation in rare subgroups.

Moreover, these location-specific differences within the ESD subgroup are also technically plausible. Technical success was lowest in duodenal ESD (83.3%), whereas it was higher in gastric ESD (97.5%) and left hemicolon ESD (93.8%), and reached 100% in right hemicolon and rectal ESD. This pattern may reflect the particular technical demands of duodenal closure, where the narrow and angulated lumen, thin wall, frequent tangential scope positioning, and the generally higher vulnerability to tension-related delayed adverse events can make controlled bite placement and approximation more difficult. By contrast, gastric defects often provide a larger working space for needle orientation, bite placement, and thread handling; however, this advantage is not uniform across all gastric locations. In particular, G-POEM closure in the antral/pyloric region showed a lower size-adjusted suturing speed than ESD (median 0.63 vs. 1.60 mm/min), suggesting that dynamic motility, local tissue strain, and less stable scope positioning may still make closure technically demanding despite the relatively standardized mucosotomy geometry. Rectal closure may benefit from a comparatively straight access route and greater scope stability, which is in line with the high technical success observed in rectal ESD (100%). In colonic ESD, technical performance may additionally be influenced by looping, unstable scope position, and segmental angulation, which could contribute to differences between right-sided and left-sided colonic lesions. Although these anatomical and biomechanical considerations fit the observed pattern, the subgroup sizes remain limited and do not allow firm mechanistic conclusions.

Beyond feasibility, we aimed to characterize efficiency in a clinically interpretable manner. Procedure time alone is difficult to compare across heterogeneous defect sizes; therefore, time adjustment to defect size was used as an adjunct efficiency metric (mm/min). Overall, the mm/min distribution suggested that closure efficiency was generally favorable, while still demonstrating the expected heterogeneity across procedure types. Notably, size-adjusted suturing speed was descriptively lower for G-POEM than for ESD, suggesting that indication-specific technical factors – such as the dynamic antral/pyloric location and the precision required for mucosal entry closure – may influence suturing efficiency beyond defect size alone.

In the pooled cohort, defect size did not predict suturing time (R ^2^ = 0.0001572), indicating that suturing time was not explained by defect size alone and may be influenced by factors such as access, geometry, tissue tension, workflow, and procedural context.

Adverse events were uncommon, and procedure-related adverse events were rare. Most events were graded as mild or moderate according to the ASGE lexicon. The observed procedure-related short-term adverse event rate was low in this registry. However, safety interpretation remains limited by the retrospective design, lack of standardized post-discharge follow-up, and the occurrence of one severe delayed bleeding event after discharge. These limitations underscore the need for careful patient selection, meticulous closure technique, and appropriate post-procedural monitoring.

Endoscopic defect management encompasses a broad range of approaches. OTS suturing platforms, TTS tack-and-suture systems, TTS clips, and OTS clips differ in device setup, tissue-capture mechanism, maneuverability, and applicability to larger or tension-bearing defects. Within this landscape, EHS represents a TTS needle-based suturing concept that enables sequential tissue bites and suture-based approximation after device preparation.

Early Western experience with EHS using SutuArt (19 procedures) reported a technical success rate of 78.9%, with a median/mean operation time of around 40 minutes and a measurable reduction in stitch time 18 . Building on this early EHS experience, our European multicenter registry demonstrates numerically high primary technical success across major indications and low adverse event rates, supporting the feasibility of EHS in routine therapeutic practice, while also underscoring that outcomes were not uniform across all anatomical and procedural subgroups.

The present registry adds multicenter real-world data on EHS across a broad range of closure scenarios, including technically demanding defects. Standard clip-based and suturing-based closure strategies remain established options for many gastrointestinal defects, and the present single-arm registry was not designed to determine comparative performance against these modalities. Further comparative studies will be needed to define more precisely how EHS performs relative to conventional clip-based closure or other suturing strategies in specific indications.

The strengths of this study include its multicenter European design, use of real-world data, predefined clinically relevant end points with standardized adverse event grading, and a comprehensive evaluation of feasibility, safety, and efficiency including size-adjusted metrics. Moreover, this study reports the largest collection of EHS cases from outside Japan.

This study has several limitations. First, it was a retrospective, multicenter, single-arm registry without a standardized or randomized allocation strategy; EHS use was at the investigator’s discretion, and no denominator of all eligible defects or comparable cases treated with alternative closure methods was available. Therefore, selection bias cannot be excluded, and it remains unclear whether EHS was preferentially used for technically favorable defects or for more complex defects in which clip-based closure was considered inadequate. Second, the registry included heterogeneous indications, anatomical locations, and defect morphologies, ranging from post-resection defects to mucosotomy/tunnel-entry closures and a small number of clinically distinct rescue indications, limiting the generalizability of pooled estimates. Third, in-hospital clinical success was restricted to the index hospitalization; post-discharge events were not captured in a standardized manner across centers and were recorded only if reported back to the treating institution, limiting interpretation of safety beyond discharge. Finally, robust assessment of the learning curve was not feasible because true cumulative operator experience, standardized workflow variables, and detailed case-complexity metrics were not captured.

### Conclusion

In this European multicenter real-world registry, EHS with SutuArt showed high technical and in-hospital clinical success with a low short-term adverse event rate across major defect closure indications. These findings should be interpreted as exploratory real-world data. Future prospective comparative studies using standardized complexity metrics, timing definitions, and post-discharge follow-up are needed to define its relative performance compared with alternative closure and suturing platforms.

## Supplementary Material

Supplementary Material Zusatzmaterial
