# Supplementary material for: Technical and clinical success of endoscopic hand-suturing: a European multicenter study
Source: Endoscopy. 2026 Sep 30;58(11):1199–210. doi: 10.1055/a-2909-3681 (PMC13758988; doi:10.1055/a-2909-3681)
Supplement: Supplementary file 1 [file 10-1055-a-2909-3681_29594588.pdf]

## Supplementary material

## Technical and Clinical Success of Endoscopic Hand-Suturing: A European Multicenter Study

Mousa Ayoub, Esma Z. Kizilaslan, Sandra Nagl, Fatih Aslan, Torsten Beyna, Oscar Cahyadi, Seung-Hun Chon, Werner Dolak, Jacek Drzewiecki, Peter Ewald, Stefan K. Gölder, Przemysław Kasprzyk, Arjun D. Koch, Konstantinos Kouladouros, Raúl Honrubia López, Annemijn D. I. Maan, Pedro De Maria Pallares, Khanh Do-Cong Pham, Daniel R. Quast, Pedro J. Rosón Rodríguez, Enrique Rodriguez de Santiago, Sofia P. Carbonell, Markus W. Scheppach, Michal Spychalski, Shunya Takayanagi, Christian Rafael Torres Reyes, Thomas Veiser, Benjamin M. Walter, Jochen Weigt, Christoph Zeidler, Helmut Messmann, Alanna Ebigbo.

**Table 1s** Outcomes by procedural context. Additional descriptive subgroup analyses were performed separating resection-related defects (ESD, EID, EMR, EFTR) from mucosotomy/tunnel-entry closures (G-POEM, STER). Reported outcomes include technical success, overall defect closure, in-hospital clinical success, suturing speed (mm/min), and adverse events.

| Group | Technical success, n/N (%) | Overall defect closure, n/N (%) | In-hospital clinical success, n/N (%) | Suturing speed, median (Q1–Q3) mm/min | Overall AEs, n/N (%) | Procedure-related AEs, n/N (%) |
|---|---|---|---|---|---|---|
| Resection-related defects (ESD, EID, EMR, EFTR) | 271/279 (97.1%; 95%CI 94.4–98.5) | 272/279 (97.5%; 95% CI 94.9–98.8) | 276/279 (98.9%; 95%CI 96.9–99.6) | 1.50 (Q1 1.08, Q3 2.77) [n=271] | 5/279 (1.8%) | 4/279 (1.4%) |
| Mucosotomy / tunnel-entry closures (G-POEM, STER) | 40/44 (90.9%; 95%CI 78.8–96.4) | 41/44 (93.2%; 95%CI 81.8–97.7) | 44/44 (100%; 95%CI 92.0–100) | 0.67 (Q1 0.47, Q3 1.00) [n=44] | 3/44 (6.8%) | 0/44 (0%) |
